# Understanding the Role of Chemokines and Cytokines in Experimental Models of Herpes Simplex Keratitis

**DOI:** 10.1155/2017/7261980

**Published:** 2017-04-09

**Authors:** Tayaba N. Azher, Xiao-Tang Yin, Patrick M. Stuart

**Affiliations:** Department of Ophthalmology, Saint Louis University, Saint Louis, MO, USA

## Abstract

Herpes simplex keratitis is a disease of the cornea caused by HSV-1. It is a leading cause of corneal blindness in the world. Underlying molecular mechanism is still unknown, but experimental models have helped give a better understanding of the underlying molecular pathology. Cytokines and chemokines are small proteins released by cells that play an important proinflammatory or anti-inflammatory role in modulating the disease process. Cytokines such as IL-17, IL-6, IL-1*α*, and IFN-*γ* and chemokines such as MIP-2, MCP-1, MIP-1*α*, and MIP-1*β* have proinflammatory role in the destruction caused by HSV including neutrophil infiltration and corneal inflammation, and other chemokines and cytokines such as IL-10 and CCL3 can have a protective role. Most of the damage results from neutrophil infiltration and neovascularization. While many more studies are needed to better understand the role of these molecules in both experimental models and human corneas, current studies indicate that these molecules hold potential to be targets of future therapy.

## 1. Introduction

Herpes simplex keratitis (HSK) is a leading cause of corneal blindness in the world [1]. Primary infection results after direct contact of mucosal membrane with herpes simplex virus-1 (HSV-1) and involves viral replication in the cornea [2, 3]. The virus then establishes latency in the trigeminal ganglia leading to recurrent infections [1, 4]. Previous studies in mouse models have shown host immune response which involves both innate and adaptive immunity including natural killer (NK) cells, dendritic cells, macrophages, neutrophils, and T cells [5, 6]. These cells are recruited to the site and activated by the release of chemokines and cytokines [6, 7].

Cytokines are small proteins released by cells that play an important proinflammatory or anti-inflammatory role in modulating the disease process. Categories of cytokines include interferons, TGF-*β*, interleukins, and chemokines [8]. Chemokines have a chemoattractant role and are divided into four families based on the location of cysteine residues in the ligands: CXC, CC, C, and CX3C [9]. CXC chemokines usually attract neutrophils, but not monocytes. In mice, CXC chemokines include macrophage inflammatory protein (MIP-2), interferon-induced protein 10 kDa (IP-10), and KC. CC chemokines, on the other hand, attract monocytes, lymphocytes, NK cells, basophils, and eosinophils. Examples of CC chemokines include macrophage inflammatory protein-1*α* (MIP-1*α*) and MIP-1*β* monocyte chemoattractant protein (MCP-1) and regulated upon activation normal T expressed and presumably secreted protein (RANTES) [10].

Herpes simplex keratitis can be divided into preclinical (week 1 postinfection) and clinical phases (10–20 days postinfection). Corneal infiltration begins in preclinical period, but maximum cloudiness does not occur until the clinical phase [11]. Immunological damage primarily results from CD4+ T lymphocytes and neutrophil migration into the cornea [3]. Leukocyte infiltration in HSK is biphasic. Among the chemokines, IP-10, MIP-2, and MCP-1 are seen during the initial period postinfection and are most likely to contribute to primary migration of leukocytes [10]. MIP-1*α* and MIP-1*β* concentrations increase shortly after the initial leukocyte infiltration. While MIP-2, MCP-1, MIP-1*α*, and MIP-1*β* are thought to play a significant role in the development of the clinical disease, KC, IP-10, and RANTES may not be directly involved in the development of HSK [10, 12]. Although numerous pathological processes occur simultaneously post-HSV infection and impact each other to cause irreversible tissue damage and scarring, we divided this review between cytokines and chemokines that cause neutrophil infiltration and cytokines and chemokines that lead to angiogenesis.

## 2. Cytokines and Chemokines Promoting Neutrophil Infiltration

While many cells infiltrate the cornea, actual damage results from invasion of neutrophils and CD4+ T cells [5, 13]. In humans, IL-8 acts as a chemoattractant for neutrophils; however, mice lack IL-8 [14]. Among other participants that promote neutrophil infiltration is IL-17. Besides acting as a chemoattractant for neutrophils, IL-17 also plays a role in the survival of neutrophils and causes cells to produce molecules such as oxy-radicals and metalloproteinases instigating tissue damage [13, 15]. In one study, mice lacking IL-17 receptor (IL-17R) had decreased severity of the lesion during the early stages of infection due to lack of response to IL-17 [16]. Mice lacking IL-17R showed negligible damage to epithelial layer, little fibrosis, and decreased infiltration of CD4+ T cells, compared to wild-type mice that had extensive fibrosis of stromal and loss of corneal epithelial layer when infected with HSV-1 [13]. Suryawanshi et al. demonstrated in their study a biphasic upregulation of IL-17 in HSK. Initial upregulation of IL-17 began on day 2 postinfection but returned to preinfection levels by day 5 around day 5; another increase in levels of IL-17 was noted starting day 7 postinfection. In early phase, *γδ* T cells were the primary producers of IL-17, while Th17 cells were the primary producers of IL-17 during the later phase most likely in response to the upregulation of IL-6 and TGF-*β* (IL-6 and TGF-*β* cause CD4+ T cells to differentiate into Th17 cells) [13]. CCL20, a chemokine, also plays a role in drawing Th17 cells towards the lesions which can further contribute to the increase of IL-17 [13]. Neutrophil infiltration in the cornea was decreased in mice lacking IL-17R and in mice where IL-17 was neutralized [13]. Hence, IL-17 most likely plays an important role in the migration of neutrophils in HSK [13, 15]. However, some studies have suggested that increase in neutrophil migration is only transient and returns to baseline 4 days postinfection [16]. Additionally, the viral titers in IL-17R-deficient mice and wild-type mice were comparable [16]. Thus, IL-17 plays no role in viral replication and only mitigates tissue damage; however, it plays a role in stimulating other proinflammatory cytokines [16].

IL-17 also induces the production of IL-6, a proinflammatory cytokine. Mice lacking IL-17R had decreased concentration of IL-6 [15]. Staats and Lausch demonstrated a significant increase of IL-1*α* and IL-6 in corneas infected with HSV compared to uninfected corneas [11]. Treating the mice with antibody treatment (to prevent HSK) decreased the production of both IL-1*α* and IL-6 [11]. IL-6 impacts corneal inflammation and neutrophil infiltration [17]. IL-6 levels increase considerably following HSV-1 infection [18]. In a study conducted by Fenton et al., mice lacking IL-6 had decreased corneal opacity two days post-HSV infection. These mice also exhibited decreased inflammation of the cornea and decreased neutrophil recruitment. Antibody targeting IL-6 in wild-type mice resulted in decreased inflammation of the cornea [17]. Similar to the effects of IL-17, the viral titers between wild-type mice and IL-6-deficient mice were not significant (*p* > 0.7). Additionally, the difference in corneal inflammation between the two groups was only transient, and it rivaled each other postinfection on day 4. Nevertheless, the administration of IL-6 with HSV-1 in mice lacking IL-6 resulted in increased concentration of macrophage inflammatory protein-2 (MIP-2) and MIP-1*α* [17]. Furthermore, mice lacking IL-6 had decreased concentration of MIP-2 [17]. Since MIP-2 and MIP-1*α* are chemoattractants for neutrophils, decreased production of IL-6 can decrease neutrophil infiltration [19]. As exhibited by Yan et al., antibody targeting IL-10 increases the production of IL-6, MIP-2, and MIP-1*α*, but the administration of IL-6 does not change the concentration of IL-10 [20]. Thus, it can be suggested that inhibition of IL-10 causes increased levels of IL-6, consequently leading to increased production of MIP-2 and MIP-1*α* which then attracts neutrophils and increases the severity of the lesion [17, 19, 20].

Anti-HSV-1 gD antibody protects the cornea against HSK; injecting this antibody results in decreased levels of proinflammatory mediators such as MIP-2, MCP-1, MIP-1*α*, and MIP-1*β*. Hence, it can be assumed that these molecules partake in HSK inflammatory response [12]. MIP-1*α* helps attract both neutrophils and T cells and is produced by numerous cells including neutrophils, macrophages, and T cells. Although virus replication was unchanged between wild-type mice and MIP-1*α*-deficient mice, the severity of the illness as measured by the growth of new blood vessels and the infiltration of inflammatory cells was decreased in mice lacking MIP-1*α*. Additionally, these MIP-1*α*-deficient mice also had lower levels of IL-2 and IFN-*γ* [12].

MIP-2 is another CXC chemokine that helps attract neutrophils to the site of lesion. In mice infected with HSV-1, MIP-2 was upregulated postinfection. Additionally, mice receiving antibody against MIP-2 had decreased corneal opacity and reduced neutrophil infiltration [19]. MIP-2 and MIP-1*α* are generated with the help of IL-1*α* and IL-6 [16]. Inducing IL-1*α* increased levels of MIP-2 and MIP-1*α*, while neutralizing IL-1*α* resulted in decreased production of MIP-2 [17, 19]. IL-17 also generates MIP-2 in addition to generating IL-6 [16]. Mice with defective IL-17 receptors had decreased production of MIP-2 and MIP-1*α* early during the infection [16]. Other molecules modulating the production of MIP-2 include other chemokines such as monocyte chemoattractant protein-1 (MCP-1). Decrease in MCP-1, a CC chemokine, resulted in increased production of MIP-2 leading to increased severity of HSK [21].

Th17 cells produce IL-17 that helps draw neutrophils towards the lesions, leading to tissue damage. IFN-*γ* on the other hand suppresses Th17 cells and IL-17 expression [13, 16]. Early depletion of IFN-*γ* increases the severity of the lesion and also leads to severe encephalitis in mice [13]. Mice lacking IFN-*γ* had elevated levels of IL-17 (*p* < 0.04), increased production of MIP-2 (*p* < 0.3), and accelerated corneal opacity [16]. Although these studies show protective role of IFN-*γ* against IL-17 in mice cornea, other studies have found IFN-*γ* to be harmful in the cornea. IFN-*γ* stimulates cytokine secretion, induces phagocytosis, activates neutrophils, and upregulates MHC expression [22]. Treatment with antibodies against IFN-*γ* and IL-2 reversed the severity of inflammation [3, 22, 23]. Furthermore, treatment with anti-IFN-*γ* antibody resulted in reduced platelet endothelial cell adhesion molecule-1 (PECAM-1) expression (PECAM-1 partakes in neutrophil extravasation) [3, 22]. Further research is needed to better understand the role of IFN-*γ* in mice infected with HSV-1.

In mice infected with HSV-1, proinflammatory cytokines such as IL-2, IFN-*γ*, and TNF-*α* were upregulated [22]. IL-2 can induce the production of IFN-*γ* and TNF-*α* along with activating neutrophils and preventing their apoptosis. Treating infected mice with antibodies against IL-2 decreased the migration of neutrophils into the tissue while promoting neutrophil apoptosis [3]. Similarly, lymphotoxin-alpha (LT-*α*), a member of TNF superfamily, produced by activated CD4+ Th cells, also has proinflammatory role in mice. Mice treated with antibodies against LT-*α* resulted in decreased severity of the lesions and decreased production of other chemokines [24].

While many of the above cytokines such as IL-17, IL-6, IL-1*α*, and IFN-*γ* and chemokines such as MIP-2, MCP-1, MIP-1*α*, and MIP-1*β* have proinflammatory role in the destruction caused by HSV-1 including neutrophil infiltration and corneal inflammation, other chemokines and cytokines such as IL-10 and CCL3 can have a protective role. In a study conducted by Yan et al., IL-10, a cytokine produced by mice corneal epithelial cells and fibroblasts, also impacted neutrophil migration and other chemokine and cytokine production [20]. Antibody neutralizing IL-10 increased the production of IL-6, MIP-2, and MIP-1*α* and worsened the severity of the lesion due to neutrophil infiltration; in line with that, mice lacking IL-10 gene exhibited increased severity of HSK. The addition of IL-6 to IL-6-deficient mice did not increase IL-10 [17, 20]. As per this study, IL-10 most likely has a protective role in mice HSK models [20]. Similarly, in a study by Tumpey et al., the administration of IL-10 resulted in the inhibition of HSK with decreased corneal opacity. It reduced the risk of blindness and neovascularization. In these mice, the concentrations of IL-2 and IL-6 were significantly reduced although no changes were noted in IL-1*α* levels [25]. Similar results were seen in other studies including decreased neutrophil infiltration after the administration of IL-10 [26]. Likewise, CCL3, a proinflammatory cytokine, has shown to have protective role in HSK. Mice lacking CCL3 had increased severity of the disease [27].

## 3. Angiogenesis and Neovascularization in HSK

Besides neutrophil infiltration and inflammation, other processes that lead to tissue damage include angiogenesis. Normal cornea is avascular; however, recurring HSK infections lead to neovascularization which causes corneal scarring and fibrosis [28, 29]. With the growth of new blood vessels, inflammatory cells have easier access to the eye [30]. Neovascularization is stimulated by vascular endothelial growth factor (VEGF) [30]. MicroRNAs (miRNAs) are gene regulators that can be induced by changes in the cellular environment. miR-132 is a microRNA that is activated in HSK and is implicated in angiogenesis [30]. Mice lacking IL-17 receptor had decreased levels of miR-132 suggesting a role of IL-17 in angiogenesis and gene regulation [30].

Normal cornea has VEGF present, but it binds to the soluble form of the VEGF receptor 1 (sVEGFR-1) preventing VEGF from acting in the cornea to create new blood vessels. Imbalance between concentrations of VEGF and sVEGFR-1 results in excess VEGF leading to angiogenesis [31]. HSV-1 infection increases the synthesis of VEGF-A, a potent inducer of new blood vessels, while decreasing the concentration of sVEFGR-1 through the production of matrix metalloproteinases such as MMP-2, MMP-7, and MMP-9 [18, 31, 32]. These matrix metalloproteinases degrade sVEGFR-1 resulting in imbalance between VEGF and sVEGFR-1 levels; this relative increase in VEGF concentration leads to neovascularization [31].

In a study by Suryawanshi et al., HSK increased IL-17 expression causing imbalance between VEGF and sVEGFR-1 concentrations. IL-17 stimulated the production of IL-6. Together, IL-17 and IL-6 enhanced VEGF-A production. Additionally IL-17 promoted MMP-2, MMP-8, and MMP-9 expressions; these matrix metalloproteinases are capable of degrading sVEGFR-1. Overall, the study exhibited that mice lacking IL-17R had decreased incidence and severity of neovascularization [15]. Similar results were seen in mice given antibodies to IL-17 [15]. Additional sources of VEGF and MMP include neutrophils. Thus, many of the molecules involved in attracting neutrophils to the site of the lesion indirectly participate in neovascularization of the cornea [15].

Likewise, IL-6, a proinflammatory cytokine produced by HSV-1-infected cells, stimulates uninfected epithelial cells in the cornea and fibroblasts in the stroma to produce VEGF [14, 18]. In a study by Biswas et al., IL-6 was injected into murine subconjunctiva which led to increased VEGF production. Additionally, VEGF levels decreased in a dose-dependent manner when antibody neutralizing IL-6 was given to mice [18]. Lastly, IL-1 is a proinflammatory cytokine produced by HSV-1-infected cells that induces corneal cells to produce VEGF and stimulate angiogenesis [18, 33]. Furthermore, IL-1 increases neutrophil infiltration into the cornea along with the concentration of MIP-2 and IL-6 [33].

## 4. Conclusion

Herpes simplex keratitis is a disease of the cornea caused by HSV-1. It is a leading cause of corneal blindness in the world [1]. Underlying molecular mechanism is still unknown, but experimental models have helped give a better understanding of the underlying molecular pathology. Cytokines and chemokines are small proteins released by cells that play an important proinflammatory or anti-inflammatory role in modulating the disease process [8]. Cytokines such as IL-17, IL-6, IL-1*α*, and IFN-*γ* and chemokines such as MIP-2, MCP-1, MIP-1*α*, and MIP-1*β* have proinflammatory role in the destruction caused by HSV-1 including neutrophil infiltration and corneal inflammation, and other chemokines and cytokines such as IL-10 and CCL3 can have a protective role. Most of the damage results from multiple actions of proinflammatory molecules, ranging from neutrophil infiltration to neovascularization. While many more studies are needed to better understand the role of these molecules in both experimental models and human corneas, current studies have promoted these molecules to be targets of future therapy.
